# A Predictive Model Based on a New CI-AKI Definition to Predict Contrast Induced Nephropathy in Patients With Coronary Artery Disease With Relatively Normal Renal Function

**DOI:** 10.3389/fcvm.2021.762576

**Published:** 2021-10-28

**Authors:** Hanjun Mo, Fang Ye, Danxia Chen, Qizhe Wang, Ru Liu, Panpan Zhang, Yaxin Xu, Xuelin Cheng, Zhendong Mei, Yan Zheng, Yuxiang Dai, Sunfang Jiang, Junbo Ge

**Affiliations:** ^1^Department of General Practice, Zhongshan Hospital, Fudan University, Shanghai, China; ^2^Department of Radiology, Zhongshan Hospital, Fudan University, Shanghai, China; ^3^State Key Laboratory of Genetic Engineering, School of Life Sciences, Human Phenome Institute, Fudan University, Shanghai, China; ^4^Department of Cardiology, Zhongshan Hospital, National Clinical Research Center for Interventional Medicine, Shanghai Institute of Cardiovascular Disease, Shanghai, China; ^5^Ministry of Education Key Laboratory of Public Health Safety, School of Public Health, Fudan University, Shanghai, China; ^6^Health Management Center, Zhongshan Hospital, Fudan University, Shanghai, China

**Keywords:** contrast induced nephropathy (CIN), coronary artery disease, contrast media (CM), incidence, risk factor, predictive model

## Abstract

**Background:** Contrast induced nephropathy (CIN) is a common complication in patients receiving intravascular contrast media. In 2020, the American College of Radiology and the National Kidney Foundation issued a new contrast induced acute kidney injury (CI-AKI) criteria. Therefore, we aimed to explore the potential risk factors for CIN under the new criteria, and develop a predictive model for patients with coronary artery disease (CAD) with relatively normal renal function (NRF).

**Methods:** Patients undergoing coronary angiography or percutaneous coronary intervention at Zhongshan Hospital, Fudan University between May 2019 and April 2020 were consecutively enrolled. Eligible candidates were selected for statistical analysis. Univariate and multivariate logistic regression analyses were used to identify the predictive factors. A stepwise method and a machine learning (ML) method were used to construct a model based on the Akaike information criterion. The performance of our model was evaluated using the area under the receiver operating characteristic curves (AUC) and calibration curves. The model was further simplified into a risk score.

**Results:** A total of 2,009 patients with complete information were included in the final statistical analysis. The results showed that the incidence of CIN was 3.2 and 1.2% under the old and new criteria, respectively. Three independent predictors were identified: baseline uric acid level, creatine kinase-MB level, and log (N-terminal pro-brain natriuretic peptide) level. Our stepwise model had an AUC of 0.816, which was higher than that of the ML model (AUC = 0.668, *P* = 0.09). The model also achieved accurate predictions regarding calibration. A risk score was then developed, and patients were divided into two risk groups: low risk (total score < 10) and high risk (total score ≥ 10).

**Conclusions:** In this study, we first identified important predictors of CIN in patients with CAD with NRF. We then developed the first CI-AKI model on the basis of the new criteria, which exhibited accurate predictive performance. The simplified risk score may be useful in clinical practice to identify high-risk patients.

## Introduction

With the application of interventional therapy in cardiovascular diseases, coronary angiography (CAG) has become the gold standard for diagnosing coronary artery disease (CAD). Percutaneous coronary intervention (PCI) has also become one of the most important treatments for patients with CAD. Regardless of the above treatments, use of contrast media (CM) is essential. However, due to the nephrotoxicity of CM, patients exposed to them may develop contrast induced nephropathy (CIN), also known as contrast induced acute kidney injury (CI-AKI). CIN is the third leading cause of hospital-acquired acute kidney injury (1). This complication prolongs the patient's hospital stay and increases medical expenses, resulting in irreversible kidney injury, need for dialysis, or even death (2). Since CIN does not have effective therapies, early identification of high-risk patients and effective interventions are extremely important.

CIN is usually defined as an increase of ≥0.5 mg/dL or ≥25% in baseline serum creatinine (SCr), within 48–72 h after exposure to CM, excluding other causes of renal function impairment (3). In January 2020, the American College of Radiology and the National Kidney Foundation jointly issued a consensus (4) and recommended that the diagnostic criteria of CA-AKI/CI-AKI be referred to the one proposed by kidney disease improving global outcomes (KDIGO), that is, when within 48 h of CM administration, the SCr increased by ≥0.3 mg/dL (26.5 μmol/L) or ≥1.5 times the baseline value (5). To date, few studies have compared the incidence of CIN based on these two diagnostic criteria, and no model has been constructed on the basis of the new criteria.

Baseline renal insufficiency is the most important risk factor for CIN, and many other risk factors, such as advanced age and diabetes, have also been recognized (6). In clinical practice, patients with chronic kidney disease (CKD) undergo hydration before and after surgery (7, 8). However, for those with relatively normal renal function (NRF), it remains unknown whether there are new, unique indicators that can predict the occurrence of CIN. Therefore, we aimed to explore the potential risk factors for CIN among patients with CAD with NRF and establish a predictive model based on new criteria.

## Methods

### Study Population

Consecutive patients undergoing CAG or PCI at Zhongshan Hospital, Fudan University, between May 2019 and April 2020, were retrospectively enrolled. Demographic data and baseline clinical characteristics were recorded, including age, sex, blood pressure, comorbidities, medical history, laboratory examinations, and procedure-associated factors. The endpoint was CIN occurrence. Inclusion criteria were patients diagnosed with CAD by CAG, with documented renal function (SCr level) at baseline and 48 h after the procedure. Exclusion criteria were patients receiving continuous dialysis for end-stage renal disease, exposure to CM within 1 week before surgery, use of nephrotoxic drugs, severe infections and liver insufficiency, combined with tumors, allergy to CM, and women during pregnancy and lactation. All included patients used low- or iso-osmolarity contrast agents and did not receive hydration therapy. The study was approved by the ethics committee of Zhongshan Hospital, Fudan University. A written informed consent was obtained from all patients upon admission, which allow for the analysis of clinical data for the purpose of scientific study.

### Clinical Definitions

“Contrast induced nephropathy (CIN)” or “Contrast induced acute kidney injury (CI-AKI)” was defined as an increase of ≥50% or 0.3 mg/dL in pre-PCI serum creatinine at 48 h after surgery (4). “Coronary artery disease (CAD)” was defined as at least one major coronary artery (left main artery, left anterior descending artery, left circumflex artery, or right coronary artery) stenosis ≥50%, confirmed by CAG (9). “Hypertension” was defined as systolic blood pressure ≥140 mmHg and/or diastolic blood pressure ≥90 mmHg, or previous diagnosis of hypertension and taking antihypertensive medications (10, 11). “Diabetes mellitus” was defined on the basis of the American Diabetes Association criteria (12). “Moderate to severe congestive heart failure (CHF)” was defined as New York Heart Association (NYHA) functional class III–IV (13). “Anemia” was defined as a hematocrit value <39% for men and <36% for women (14). Hyperuricemia was defined as fasting serum uric acid level >7.0 mg/dL in men and >6.0 mg/dL in women (15). Perioperative myocardial infarction was defined on the basis of the fourth universal definition of MI (16).

### Development of Stepwise Model and Risk Score

The data were preprocessed before the formal analysis, and missing values of eGFR were handled on the basis of the CKD-EPI formula (consistent with previously recorded data) (17). Values in the variables of “Hypertension” and “Diabetes Mellitus” were corrected on the basis of the definitions, in combination with actual blood pressure and blood glucose level (or glycosylated hemoglobin) of patients. Univariate and multivariate logistic regression analyses were used to identify the predictive factors. Variables with a *P* < 0.1 in the univariate analysis were available for further multivariate regression. A stepwise selection method was used to construct the best model on the basis of the Akaike information criterion (AIC). We also used the machine learning (ML) method (elastic net) to select statistically significant variables as a supplementary verification.

To evaluate the performance of our stepwise model, we used the area under the receiver operating characteristic curves (AUC) and calibration curves. Subsequently, to facilitate clinical use, we simplified the stepwise model and converted it into a risk score. Receiver operating characteristic (ROC) curves were used to identify the cut-off values of the continuous variables. Weighted scores were assigned to each risk factor on the basis of their new odds ratios (ORs). On the basis of their total scores, patients were further divided into different risk groups.

### Statistical Analysis

All statistical analyses and plotting were performed using R 3.6.3 (Lucent Technologies, New Providence, the United States, https://www.r-project.org) and GraphPad Prism 8 (GraphPad Software, LaJolla, CA, USA, https://www.graphpad.com/scientific-software/prism). Some of the R packages “tidyverse,” “MASS,” “pROC,” “RMS” were used. Continuous variables conforming to the normal distribution were represented as mean ± standard deviation (SD); otherwise, they are presented as median (interquartile range). Categorical variables were expressed as frequency (percentage) using the chi-square test or Fisher's exact test. All *P*-values were two-sided, and statistical significance was set at *P* < 0.05.

## Results

### Patients and Clinical Characteristics

**Figure 1:**
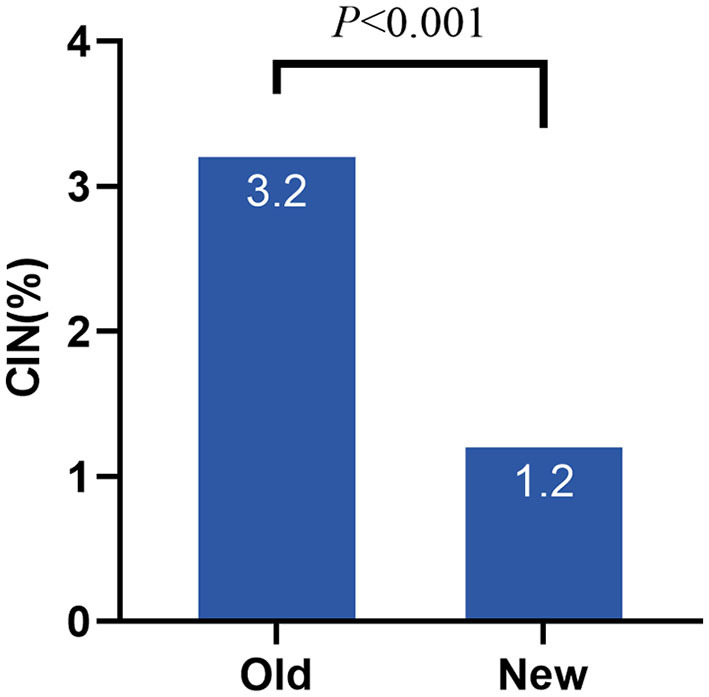
Incidence of CIN under different criteria. CIN, contrast induced nephropathy; Old, old criteria; New, new criteria.

A total of 2,383 patients with CAD after the procedure were included consecutively in this study. Some patients were excluded from the study: 41 cases without significant coronary artery lesions, 66 with a history of chronic kidney disease (CKD), 265 with eGFR <60 mL/min/1.73 m^2^, and 2 with hepatic insufficiency. In all, 2,009 patients were included in the statistical analysis. Based on the old criteria, the incidence of CIN was 3.2%, whereas it declined to 1.2% under the new criteria (Figure 1).

The baseline demographic and clinicopathologic features are listed in Table 1. Overall, the mean age of all patients was 63.3 years, and 79.0% were male. Regarding CAD subtypes and comorbidities, 22.9% had ACS, 74.4% had hypertension, and 40.9% had diabetes mellitus. Patients who underwent low-osmolarity CM accounted for 69.7% of the patients, and the mean volume of contrast was 150.17 ± 4.5 mL. Patients who developed CIN (CINs) and those who did not (non-CINs) differed substantially with respect to the following variables: diastolic blood pressure, smoking status, CAD subtype, previous acute myocardial infarction (AMI) history, and laboratory examinations (left ventricular ejection fraction, left ventricular end-systolic dimension, neutrophils, lymphocytes, neutrophil to lymphocyte ratio, eosinophils, platelet distribution width, aspartate aminotransferase [AST], low density lipoprotein, non-high-density lipoprotein, apolipoprotein B, creatine kinase-MB [CK-MB], high-sensitivity C-reactive protein, cardiac troponin T [cTnT], and N-terminal pro-brain natriuretic peptide [NT-proBNP] levels).

**Table 1:** Basic characteristics of the CIN and non-CIN groups.

	**Total** **(***n*** = 2,009)**	**CIN** **(***n*** = 24)**	**Non-CIN** **(***n*** = 1,985)**	***P*** **-value**
**Demographics and clinical characteristics**				
Age, years	63.3 (±9.9)	62.9 (±11.0)	63.3 (±9.8)	0.82
Sex				
Male	1,588 (79.0%)	22 (91.7%)	1,566 (78.9%)	0.20
Female	421 (21.0%)	2 (8.3%)	419 (21.1%)	
BMI	25.1 (±3.0)	25.0 (±3.0)	25.1 (±3.0)	0.76
Missing	45 (2.2%)	1 (4.2%)	44 (2.2%)	
Systolic blood pressure, mmHg	133.7 (±19.2)	140.2 (±19.6)	133.6 (±19.1)	0.17
Missing	6 (0.3%)	0 (0%)	6 (0.3%)	
Diastolic blood pressure, mmHg	78.6 (±10.9)	84.9 (±13.1)	78.6 (±10.8)	**0.018**
Missing	6 (0.3%)	0 (0%)	6 (0.3%)	
Heart rate, per min	76.2 (±11.7)	82.5 (±19.3)	76.1 (±11.6)	0.063
Missing	3 (0.1%)	0 (0%)	3 (0.2%)	
Smoking				
Never	1,082 (53.9%)	10 (41.7%)	1,072 (54.0%)	**0.049**
Former	378 (18.8%)	2 (8.3%)	376 (18.9%)	
Current	547 (27.2%)	12 (50.0%)	535 (27.0%)	
Missing	2 (0.1%)	0 (0.0%)	2 (0.1%)	
CAD subtype				
Stable CAD	1,548 (77.1%)	11 (45.8%)	1,537 (77.4%)	**<0.001**
ACS	461 (22.9%)	13 (54.2%)	448 (22.6%)	
NYHA classification				
I	1,818 (90.5%)	19 (79.2%)	1,799 (90.6%)	0.06
II	160 (8.0%)	3 (12.5%)	157 (7.9%)	
III	29 (1.4%)	2 (8.3%)	27 (1.4%)	
IV	2 (0.1%)	0 (0.0%)	2 (0.1%)	
**Comorbidities**				
Hypertension	1,495 (74.4%)	16 (66.7%)	1,479 (74.5%)	0.36
Diabetes mellitus	822 (40.9%)	9 (37.5%)	813 (41.0%)	0.84
Dyslipidemia				
No	1,724 (85.8%)	21 (87.5%)	1,703 (85.8%)	0.93
Hypercholesterolemia	148 (7.4%)	2 (8.3%)	146 (7.4%)	
Hypertriglyceridemia	42 (2.1%)	0 (0.0%)	42 (2.1%)	
Combined	95 (4.7%)	1 (4.2%)	94 (4.7%)	
Previous AMI	216 (10.8%)	6 (25.0%)	210 (10.6%)	**0.037**
Previous PCI	676 (33.6%)	6 (25.0%)	670 (33.8%)	0.51
Previous CABG	31 (1.5%)	1 (4.2%)	30 (1.5%)	0.31
Family history of CAD	95 (4.7%)	0 (0.0%)	95 (4.8%)	0.63
**Prior medication use**				
Anti-platelet	1,718 (85.5%)	19 (79.2%)	1,699 (85.6%)	0.37
Aspirin	1,582 (78.7%)	18 (75.0%)	1,564 (78.8%)	0.62
Clopidogrel	799 (39.8%)	10 (41.7%)	789 (39.7%)	0.84
Missing	4 (0.2%)	0 (0.0%)	4 (0.2%)	
Statins	1,565 (77.9%)	18 (75.0%)	1,547 (77.9%)	0.63
Missing	9 (0.4%)	0 (0.0%)	9 (0.5%)	
ACEI/ARB	955 (47.5%)	10 (41.7%)	945 (47.6%)	0.68
Beta-blockers	952 (47.4%)	12 (50.0%)	940 (47.4%)	0.84
Calcium channel blockers	586 (29.2%)	6 (25.0%)	580 (29.2%)	0.82
Nitrates	743 (37.0%)	9 (37.5%)	734 (37.0%)	1.0
Diuretics	161 (8.0%)	2 (8.3%)	159 (8.0%)	1.0
**Laboratory data**				
LVEF, %	61.9 (±7.6)	56.2 (±10.6)	62.0 (±7.6)	**0.003**
Missing	78 (3.9%)	0 (0%)	78 (3.9%)	
LVESD, mm	31.5 (±5.4)	35.4 (±9.7)	31.5 (±5.3)	**0.029**
Missing	78 (3.9%)	0 (0%)	78 (3.9%)	
LVEDD, mm	47.9 (±5.3)	49.2 (±7.0)	47.9 (±5.3)	0.75
Missing	78 (3.9%)	0 (0%)	78 (3.9%)	
RBC, × 10^12^ cells per L	4.4 (±0.5)	4.4 (±0.7)	4.4 (±0.5)	0.58
Hemoglobin, g/L	135.9 (±14.5)	137.0 (±20.1)	135.9 (±14.5)	0.60
Hematocrit, %	40.4 (±4.0)	40.3 (±5.4)	40.4 (±4.0)	0.86
WBC, × 10^9^ cells per L	6.7 (±1.9)	7.6 (±2.4)	6.7 (±1.9)	0.098
Neutrophils, %	61.2 (±9.4)	67.9 (±13.7)	61.1 (±9.3)	**0.002**
Lymphocytes, %	27.5 (±8.3)	21.5 (±11.1)	27.6 (±8.2)	**0.002**
NLR	2.7 (±2.3)	5.3 (±5.7)	2.7 (±2.2)	**0.002**
Eosinophils, %	2.6 (±2.2)	1.9 (±1.8)	2.6 (±2.2)	**0.041**
RDW, %	12.6 (±0.8)	12.8 (±1.0)	12.6 (±0.8)	0.29
PDW, %	13.1 (±2.6)	12.1 (±2.5)	13.1 (±2.6)	**0.036**
Missing	14 (0.7%)	0 (0%)	14 (0.7%)	
Albumin, g/L	42.3 (±3.8)	41.9 (±4.4)	42.3 (±3.8)	0.44
Missing	2 (0.1%)	1 (4.2%)	1 (0.1%)	
ALT, U/L	27.9 (±19.7)	32.7 (±17.8)	27.9 (±19.7)	0.086
Missing	2 (0.1%)	1 (4.2%)	1 (0.1%)	
AST, U/L	26.9 (±24.8)	57.7 (±77.5)	26.5 (±23.4)	** <0.001**
Missing	2 (0.1%)	1 (4.2%)	1 (0.1%)	
Baseline urea, mmol/L	5.8 (±1.6)	5.9 (±2.0)	5.8 (±1.6)	0.62
Baseline creatinine, μmol/L	78.9 (±14.9)	79.5 (±17.3)	78.9 (±14.9)	0.94
Baseline eGFR, mL/(min·1.73 m^2^)	85.2 (±13.0)	85.8 (±14.0)	85.2 (±13.0)	0.68
Baseline UA, μmol/L	343.9 (±87.1)	380.2 (±112.2)	343.5 (±86.7)	0.20
FBG, mmol/L	7.2 (±3.3)	7.2 (±2.8)	7.2 (±3.3)	0.61
Missing	28 (1.4%)	1 (4.2%)	27 (1.4%)	
HbA1c, %	6.5 (±1.3)	6.2 (±0.9)	6.5 (±1.3)	0.49
Missing	91 (4.5%)	3 (12.5%)	88 (4.4%)	
TC, mmol/L	3.7 (±1.1)	4.1 (±1.0)	3.7 (±1.1)	0.055
Missing	6 (0.3%)	0 (0%)	6 (0.3%)	
TG, mmol/L	1.9 (±1.4)	1.6 (±0.7)	1.9 (±1.4)	0.29
Missing	6 (0.3%)	0 (0%)	6 (0.3%)	
LDL, mmol/L	1.9 (±1.0)	2.3 (±1.0)	1.9 (±1.0)	**0.015**
Missing	6 (0.3%)	0 (0%)	6 (0.3%)	
NHDL, mmol/L	2.7 (±1.1)	3.0 (±1.0)	2.7 (±1.1)	**0.041**
Missing	6 (0.3%)	0 (0%)	6 (0.3%)	
HDL, mmol/L	1.0 (±0.3)	1.0 (±0.3)	1.0 (±0.3)	0.84
Missing	6 (0.3%)	0 (0%)	6 (0.3%)	
Apo A-I, g/L	1.2 (±0.2)	1.2 (±0.2)	1.2 (±0.2)	0.90
Missing	7 (0.3%)	0 (0%)	7 (0.4%)	
Apo B, g/L	0.7 (±0.2)	0.8 (±0.2)	0.7 (±0.2)	**0.025**
Missing	7 (0.3%)	0 (0%)	7 (0.4%)	
Apo E, mg/L	38.8 (±16.7)	37.4 (±11.4)	38.8 (±16.7)	0.99
Missing	7 (0.3%)	0 (0%)	7 (0.4%)	
Lipoprotein, nmol/L	105.5 (±192.4)	116.4 (±257.8)	105.3 (±191.6)	0.47
Missing	7 (0.3%)	0 (0%)	7 (0.4%)	
CK-MB, U/L	18.5 (±28.0)	67.5 (±113.1)	17.9 (±24.7)	**0.025**
Missing	10 (0.5%)	0 (0%)	10 (0.5%)	
hs-CRP, mg/L	4.4 (±10.5)	6.9 (±9.9)	4.4 (±10.5)	**0.026**
Missing	19 (0.9%)	0 (0%)	19 (1.0%)	
cTnT, ng/mL	0.1 (±0.7)	0.9 (±2.1)	0.1 (±0.6)	**<0.001**
NT-proBNP, pg/mL	369.8 (±743.1)	1,372.1 (±1,690.6)	357.7 (±716.5)	**<0.001**
Missing	4 (0.2%)	0 (0%)	4 (0.2%)	
**Procedural characteristics**				
Contrast agent				
low-osmolarity	1,401 (69.7%)	20 (83.3%)	1,381 (69.6%)	0.18
iso-osmolarity	608 (30.3%)	4 (16.7%)	604 (30.4%)	
Contrast volume, mL	150.1 (±74.5)	167.1 (±84.0)	149.9 (±74.3)	0.42
Missing	24 (1.2%)	0 (0%)	24 (1.2%)	
Infarct-related artery, *N* (%)				
LM	214 (10.7%)	2 (8.3%)	212 (10.7%)	1.0
LAD	1,670 (83.1%)	19 (79.2%)	1,651 (83.2%)	0.58
LCX	1,224 (60.9%)	14 (58.3%)	1,210 (61.0%)	0.83
RCA	1,262 (62.8%)	15 (62.5%)	1,247 (62.8%)	1.0
No. of diseased vessel, *N* (%)				
1-vessel	565 (28.1%)	8 (33.3%)	557 (28.1%)	0.93
2-vessel	603 (30.0%)	6 (25.0%)	597 (30.1%)	
3-vessel	712 (35.4%)	9 (37.5%)	703 (35.4%)	
4-vessel	129 (6.4%)	1 (4.2%)	128 (6.4%)	
No. of stents used	1.4 (±1.0)	1.4 (±1.1)	1.4 (±1.0)	0.68
Total stent length, mm	40.7 (±30.5)	37.4 (±33.8)	40.8 (±30.5)	0.37
Missing	2 (0.1%)	0 (0%)	2 (0.1%)	

*Continuous variables are shown as Mean (SD), and categorical variables are shown as frequency (percentage)*.

*Boldface represents P < 0.05*.

*BMI, body mass index; CAD, coronary artery disease; ACS, acute coronary syndrome; NYHA, New York Heart Association; AMI, acute myocardial infarction; PCI, percutaneous coronary intervention; CABG, coronary artery bypass grafting; ACEI, angiotensin converting enzyme inhibitor; ARB, angiotensin receptor blocker; LVEF, center ventricular ejection fraction; LVESD, center ventricular end-systolic dimension; LVEDD, center ventricular end-diastolic dimension; RBC, red blood cell; WBC, white blood cell; NLR, neutrophil to lymphocyte ratio; RDW, red cell distribution width; PDW, platelet distribution width; ALT, alanine transaminase; AST, aspartate aminotransferase; eGFR, estimated glomerular filtration rate calculated by the CKD-EPI equation; UA, uric acid; FBG, fasting blood glucose; HbA1c, glycosylated hemoglobin; TC, total cholesterol; TG, triglycerides; LDL, low-density lipoprotein cholesterol; NHDL, non-high-density lipoprotein cholesterol; HDL, high-density lipoprotein cholesterol; Apo A-I, apolipoprotein A1; Apo B, apolipoprotein B; Apo E, apolipoprotein E; CK-MB, creatine kinase-MB; hs-CRP, high-sensitivity C-reactive protein; cTnT, cardiac troponin T; NT-proBNP, N-terminal pro-brain natriuretic peptide; LM, center main coronary artery; LAD, center anterior descending; LCX, center circumflex artery; RCA, right coronary artery*.

### Univariate and Multivariate Analysis

All the variables in Table 1 were used in the subsequent univariate analysis, and some skewed variables (for example, alanine transaminase [ALT] and AST) were log-transformed. Table 2 shows the factors with a *P* < 0.1. A total of 22 variables were significantly associated with the development of CIN, namely vital signs, medical history, comorbidities, cardiac ultrasound, and laboratory indexes. Afterward, considering the collinearity of some existing variables, we retained systolic blood pressure and LVEF instead of diastolic blood pressure and LVESD. A stepwise logistic regression was performed to construct the model. The final multivariate results are shown in Table 3. Baseline uric acid (UA), CK-MB, and log (NT-proBNP) levels were identified as independent predictors of CIN. The AIC of the model was 213.63 (Table 3).

**Table 2:** Univariate analysis in our cohort.

**Variables**	**OR**	**95% CI**	***P*** **-value**
Systolic blood pressure, / 10 mmHg	1.19	0.97–1.45	0.091
Diastolic blood pressure, / 10 mmHg	1.68	1.17–2.40	0.005
Heart rate, per min	1.04	1.01–1.07	0.007
Smoking (current smokers)	2.71	1.20–6.13	0.016
Previous AMI history	2.82	1.01–6.80	0.030
ACS	4.05	1.80–9.29	0.001
Congestive heart failure (NYHA III-IV)	6.13	0.95–22.18	0.017
LVEF, %	0.94	0.90–0.97	0.000
LVEF <45 %	4.13	1.18–11.21	0.011
LVESD, mm	1.08	1.03–1.12	0.001
WBC, × 10^9^ cells per L	1.22	1.02–1.43	0.020
NLR	1.14	1.07–1.21	0.000
PDW, %	0.83	0.67–1.00	0.065
Log (AST), U/L	3.39	1.96–5.57	0.000
Baseline UA, μmol/L	1.00	1.00–1.01	0.040
TC, mmol/L	1.28	0.93–1.64	0.083
LDL, mmol/L	1.39	1.01–1.78	0.018
NHDL, mmol/L	1.29	0.93–1.67	0.085
Apo B, g/L	3.62	0.84–12.39	0.059
CK-MB, U/L	1.01	1.01–1.02	0.000
cTnT, ng/mL	1.49	1.19–1.80	0.000
Log (NT-proBNP), pg/mL	2.04	1.54–2.74	0.000

*Variables with P < 0.1 are included in the table*.

*OR, odds ratio; CI, confidence interval; AMI, acute myocardial infarction; ACS, acute coronary syndrome; NYHA, New York Heart Association; LVEF, left ventricular ejection fraction; LVESD, left ventricular end-systolic dimension; WBC, white blood cell; NLR, neutrophil to lymphocyte ratio; PDW, platelet distribution width; AST, aspartate aminotransferase; UA, uric acid; TC, total cholesterol; LDL, low-density lipoprotein cholesterol; NHDL, non-high-density lipoprotein cholesterol; Apo B, apolipoprotein B; CK-MB, creatine kinase-MB; cTnT, cardiac troponin T; NT-proBNP, N-terminal pro-brain natriuretic peptide*.

**Table 3:** Multivariate analysis and the independent predictors in our cohort.

**Variables**	**Model coefficient**	**OR**	**95% CI**	***P*** **-value**
Baseline UC	0.005	1.00	1.00–1.01	0.040
CK-MB, U/L	0.011	1.01	1.01–1.02	<0.001
Log (NT-proBNP), pg/mL	0.661	1.94	1.45–2.63	<0.001

*AIC = 213.63*.

*OR, odds ratio; CI, confidence interval; UA, uric acid; CK-MB, creatine kinase MB; NT-proBNP, N-terminal pro-brain natriuretic peptide*.

### Performance of the Stepwise Model

**Figure 2:**
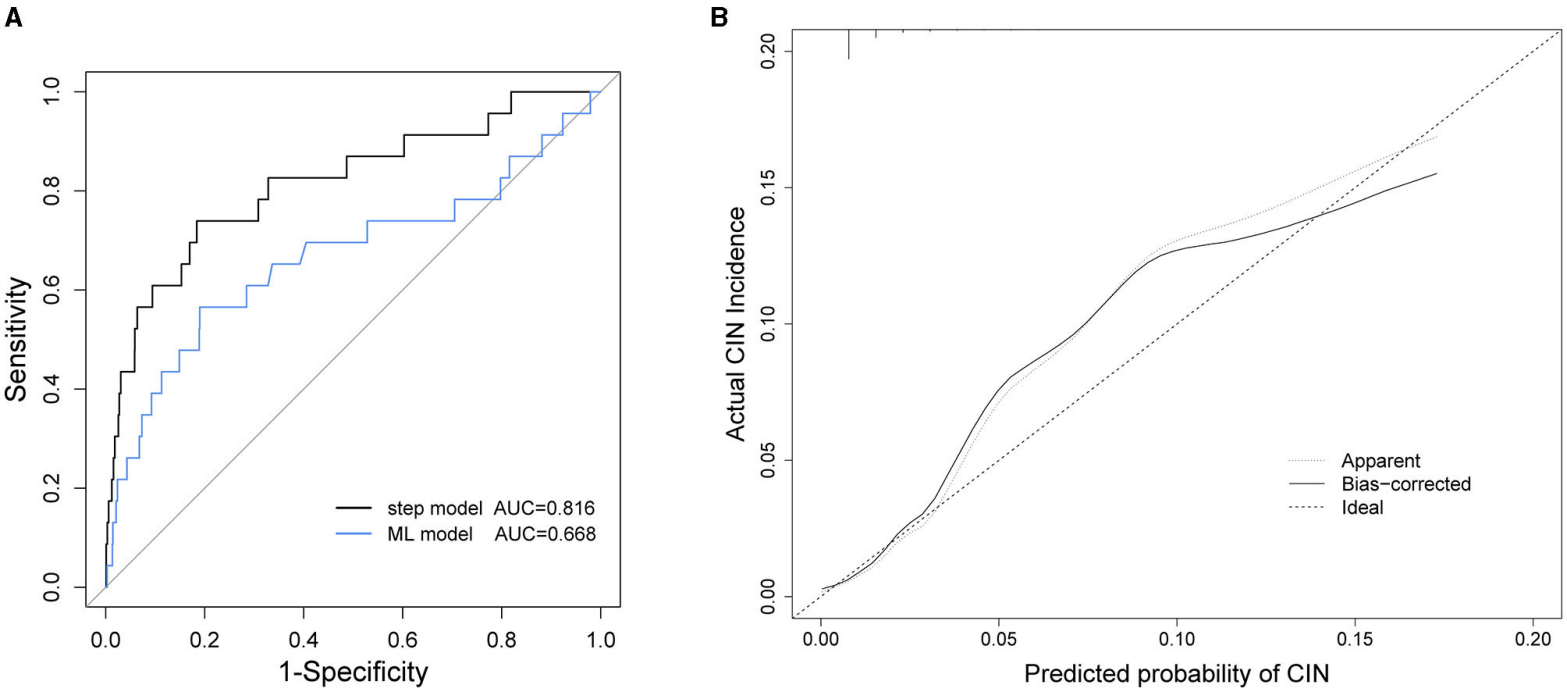
**(A)** Receiver operating characteristic (ROC) curves of stepwise model and machine learning (ML) model. **(B)** Calibration curve of stepwise model. AUC, area under the receiver operating characteristic curves; CIN, contrast induced nephropathy.

The performance of our stepwise model was evaluated using the AUC and the calibration curve (Figure 2). The AUC of the stepwise model was compared with that of the machine learning (ML) model, and the calibration was performed using internal validation. The AUC of the stepwise model was 0.816, which was higher than that of the ML model (AUC = 0.668, *P* = 0.09, Figure 2A). The calibration curve showed that the predictive incidence was significantly associated with the actual probability of CIN (Figure 2B).

### Simplified Risk Score Development

**Figure 3:**
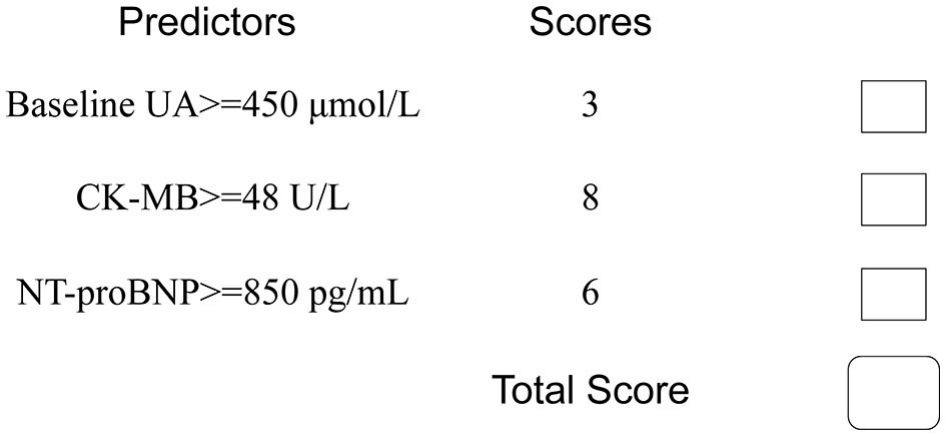
Simplified predictive score for CIN. UA, uric acid; CK-MB, creatine kinase MB; NT-proBNP, N-terminal pro-brain natriuretic peptide.

**Figure 4:**
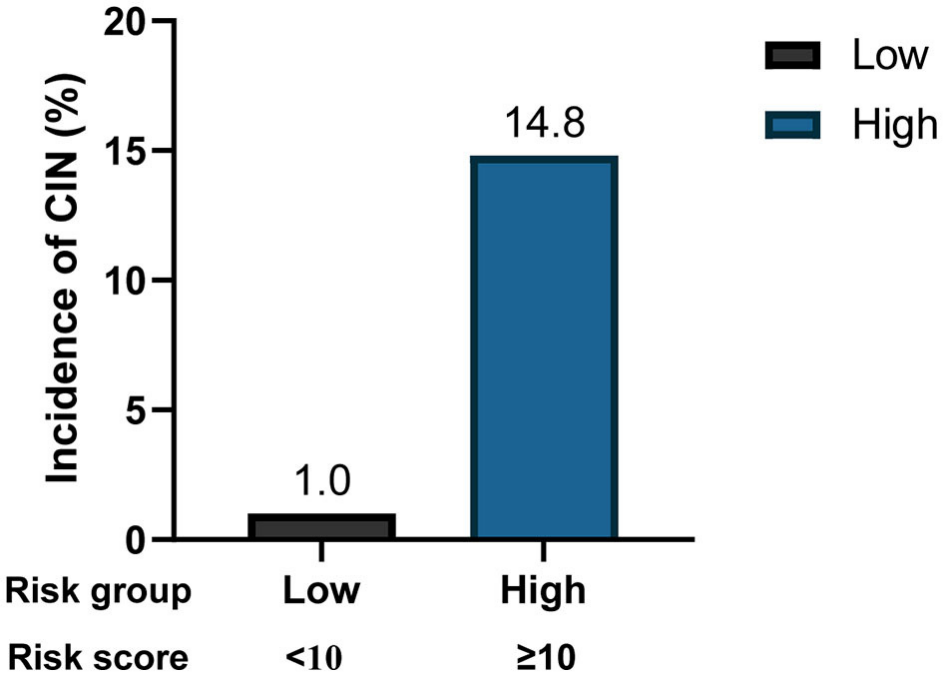
CIN incidence based on different risk groups. CIN, contrast induced nephropathy.

To facilitate implementation in clinical practice, we simplified the stepwise model to a risk score. The cut-off values of the three variables were identified using ROC curves (Supplementary Figure 1). We chose baseline UA ≥450 μmol/L, CK-MB ≥48 U/L, and NT-proBNP ≥850 pg/mL as the cut-off values, and the new multivariate results are shown in Supplementary Table 1. A weighted score of 1 was assigned to each OR value, and the final risk score is shown in Figure 3. The total scores of the patients in our cohort were between 0 and 17 points. Based on the total scores, we divided patients into two risk groups (low risk and high risk, corresponding to a total score of <10 and ≥10, respectively). The CIN incidence of patients in the low-risk group was 1.0%, while the incidence increased to 14.8% when the total score was ≥10 (Figure 4).

## Discussion

In this study, we first compared the incidence of CIN using two different criteria. The incidence of CIN was generally low, with 3.4% under the old criteria and 1.6% under the new criteria. The incidence under the old criteria was very similar to the results of Rihal et al. (18). Therefore, based on the KDIGO criteria, the incidence of CIN decreased dramatically, indicating that the old definition of CIN, to some extent, may have exaggerated the development of CI-AKI. In addition, other factors such as the different study populations and the nature of the CAG procedure may also lead to the variance of incidence rate of CIN (19).

Next, we explored the risk factors for CIN development. The results showed that, except for traditional risk factors, there were other predictors of great importance for patients with relatively NRF. In the multivariate logistic regression analysis, we determined the predictive value of baseline UA, CK-MB, and log (NT-proBNP) levels. A stepwise model and a risk score were then constructed, and to the best of our knowledge, these two are the first CIN predictive models based on the new KDIGO criteria. Our model exhibited good predictive ability, with an AUC of 0.816. Furthermore, we simplified the model to a risk score, which facilitates its use by clinicians. Two risk groups were further defined: low-risk (<10 points) and high-risk (≥10 points). The incidence of CIN increased significantly as the score increased (Figure 4).

Hyperuricemia (or baseline UA level) has been widely confirmed to influence the occurrence of CIN (20). In the present study, baseline UA level was an independent risk factor (OR, 1.004; *P* = 0.04). Similarly, other studies have proved that hyperuricemia is significantly associated with a higher risk of CI-AKI, regardless of renal function (15, 16). Nevertheless, it remains unknown whether UA lowering therapy can effectively reduce the incidence of CIN. A randomized controlled trial explored the role of allopurinol in preventing CIN after PCI and failed to show its efficacy (21). At any rate, it warned us that patients with high UA levels before PCI have the potential to develop CIN.

CK-MB is an early marker of myocardial injury with high specificity. Our analysis showed that CK-MB has an important value in predicting CIN (OR: 1.01, 95% CI: 1.01–1.02, *P* < 0.001). Previously, Zbierska-Rubinkiewicz et al. found that increased baseline CK-MB level was an independent risk factor for CIN among patients treated with PCI (*P* = 0.001) (22). The prediction model constructed by Gurm et al. also incorporated CK-MB as an important predictor (23). These findings are consistent with our results. Regarding the reason why cTnT was excluded from the model, we believe that this is due to the fact that the level of CK-MB is less affected by renal function; therefore, it can reflect patients' myocardial ischemia to a large extent. This makes CK-MB more favorable for predicting CIN.

Another finding in our study pertained to the level of NT-proBNP. In multivariate analysis, log (NT-proBNP) was statistically significant (OR, 1.94; 95%CI: 1.45–2.63, *P* < 0.001). BNP plays an important role in maintaining the circulating blood volume and osmotic pressure. In contrast, NT-proBNP, the precursor of BNP, is an important biomarker for the diagnosis of heart failure due to its long half-life and good *in vitro* stability (24). Nevertheless, since NT-proBNP is mainly filtered and cleared by the glomerulus, it is greatly affected by renal function. NT-proBNP has shown a predictive value for CIN in both STEMI and elective surgery patients (25–27). In 2015, Liu et al. found that preprocedural NT-proBNP levels could predict CIN as effectively as the Mehran risk score (13, 27). Our results are consistent with their work. When developing the simple risk score, we identified a cut-off value of NT-proBNP as ≥850 pg/mL and incorporated it into the model to achieve better prediction.

It is not clear why elevated NT-proBNP levels are associated with a higher risk of CIN. Some studies have proposed that BNP reduces the effects of catecholamines and potentiates the generation of nitric oxide, thereby potentially resulting in systemic vasodilation and renal hypoperfusion (27). Future research may further explore this potential mechanism.

Other recognized risk factors include advanced age, diabetes, CHF (NYHA III-IV), and anemia. In particular, Mehran et al. published a risk score that included eight variables: hypotension, IABP, CHF, CKD, diabetes, age >75 years, anemia, and volume of contrast (13). None of these variables showed statistical significance in our cohort, indicating that the three predictors (levels of UA, CK-MB, and NT-proBNP) were more significant. The type and amount of contrast medium are also associated with the development of CIN. It has been reported that the effect of the volume of contrast and CIN was dose dependent. In our study, the effect of contrast volume was not statistically significant (OR: 1.002, *P* = 0.256). We speculated that the amount of CM had little effect on patients with relatively NRF. Additionally, compared with studies conducted in Western countries, a smaller average amount of CM in our study (150.17 ± 4.5 mL) may also make the effect insignificant.

Our study is a well-designed research exploring the risk factors for predicting CIN in Chinese people, with a large sample size. The established stepwise model exhibited outstanding performance (AUC = 0.816) and could predict CIN precisely (Figure 2). The limitations of our work lie in the following aspects. First, the number of patients with CIN was relatively small. In other words, the incidence of CIN in our cohort was below average (new criteria: 1.2%). This may add some difficulty in determining the effects of the predictors. Moreover, the study was retrospective and could not control for all confounding factors. Finally, we used internal validation to evaluate the model in order to make full use of the data to construct the model. Therefore, additional external validation is required for further studies and model evaluation.

## Conclusions

In the present study, we first identified three independent risk factors in patients with CAD with relatively NRF: baseline UA level, CK-MB level, and NT-proBNP level of CIN. Meanwhile, we developed the first stepwise model and risk score based on the new CI-AKI criteria, which exhibited accurate predictive ability. Two risk groups were defined on the basis of the total score of the patients. This simplified risk score may be helpful in clinical practice to identify high-risk patients in the future.

## Data Availability Statement

The raw data supporting the conclusions of this article will be made available by the authors, without undue reservation.

## Ethics Statement

The studies involving human participants were reviewed and approved by the Ethics Committee of Zhongshan Hospital Fudan University (B2021-219). The patients/participants provided their written informed consent to participate in this study.

## Author Contributions

HM, FY, YD, SJ, and JG conceived and designed the study. HM, FY, ZM, YZ, and YD conducted the statistical analyses. HM and SJ drafted the manuscript. SJ and YD had full access to all the data and took responsibility for the integrity of the data and the accuracy of the data analysis. All authors participated in the interpretation of the results, revised the manuscript, read, and approved the final manuscript.

## Funding

This study was supported by the National Key Research and Development Program of China (Grant No. 2016YFC1301200), the Program for Shanghai Outstanding Medical Academic Leader (Grant No. 2019LJ15), the Scientific Research Project of Shanghai Science and Technology Commission (Grant No. 17ZR1404900), Clinical Research Plan of SHDC (Grant No. SHDC2020CR1007A), Animal Model Project of Shanghai Scientific Committee (Grant No. 19140900900), Natural Science Foundation of Shanghai (Grant No. 20ZR1439700), and Exploratory Clinical Research Projects of National Clinical Research Center for Interventional Medicine (Grant No. 2021-002). The funding agencies had no role in the design and conduct of the study, in the collection, management, analysis, interpretation of the data or in the preparation, review or approval of the manuscript, or decision to submit for publication.

## Conflict of Interest

The authors declare that the research was conducted in the absence of any commercial or financial relationships that could be construed as a potential conflict of interest.

## Publisher's Note

All claims expressed in this article are solely those of the authors and do not necessarily represent those of their affiliated organizations, or those of the publisher, the editors and the reviewers. Any product that may be evaluated in this article, or claim that may be made by its manufacturer, is not guaranteed or endorsed by the publisher.
