# Supplementary material for: A Predictive Model Based on a New CI-AKI Definition to Predict Contrast Induced Nephropathy in Patients With Coronary Artery Disease With Relatively Normal Renal Function
Source: Front Cardiovasc Med. 2021 Oct 28;8:762576. doi: 10.3389/fcvm.2021.762576 (PMC8581221; doi:10.3389/fcvm.2021.762576)
Supplement: Supplementary file 1 [file Data_Sheet_1.docx]

**Supplementary Table 1.** Simplified multivariate logistic regression analysis

| Variables | Model Coefficient | OR | 95% CI | *P*-value |
| --- | --- | --- | --- | --- |
| Baseline UA ≥450 μmol/L | 1.104 | 3.02 | 1.04-7.76 | 0.029 |
| CK-MB ≥48 U/L | 2.172 | 8.78 | 3.04-23.26 | <0.001 |
| NT-proBNP ≥850 pg/mL | 1.860 | 6.42 | 2.63-15.62 | <0.001 |

AIC = 215.01

Abbreviations: OR, odds ratio; CI, confidence interval; UA, uric acid; CK-MB, creatine kinase MB; NT-proBNP, N-terminal pro-brain natriuretic peptide.


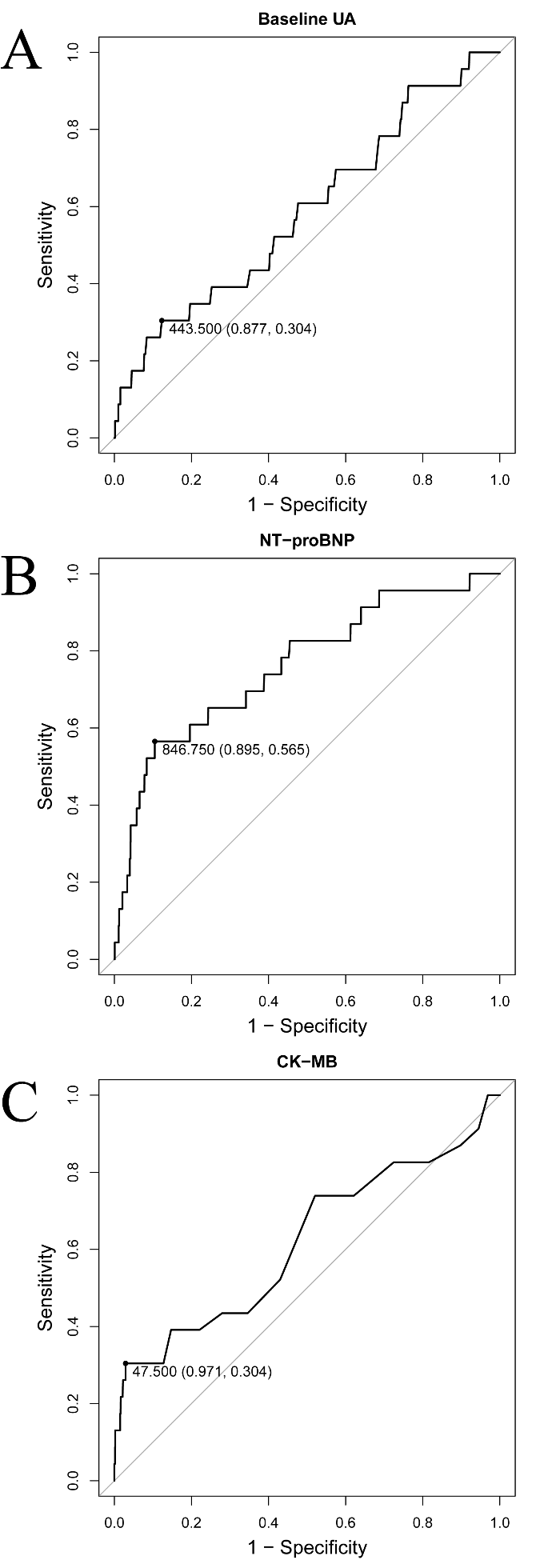
**Supplementary Figure 1.** Cut-off values of baseline UA, CK-MB, and NT-proBNP

Abbreviations: UA, uric acid; CK-MB, creatine kinase MB; NT-proBNP, N-terminal pro-brain natriuretic peptide.
